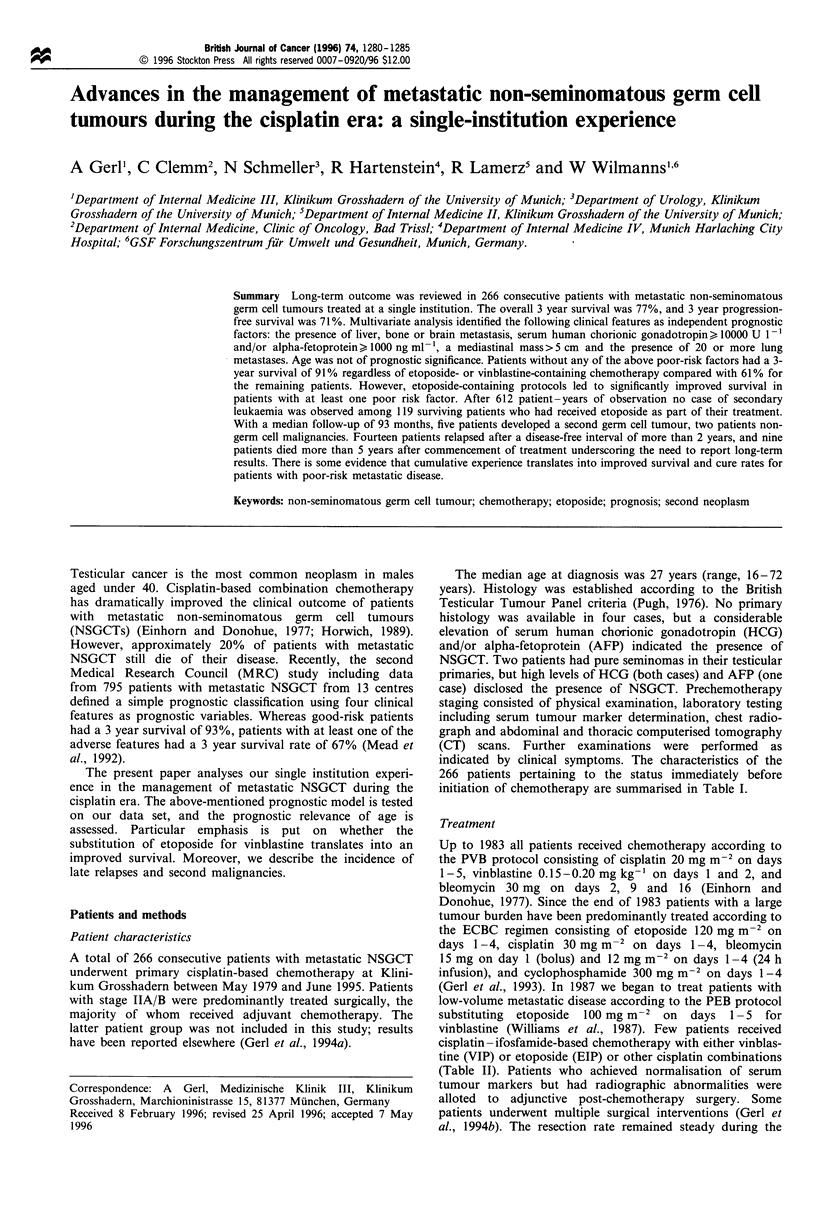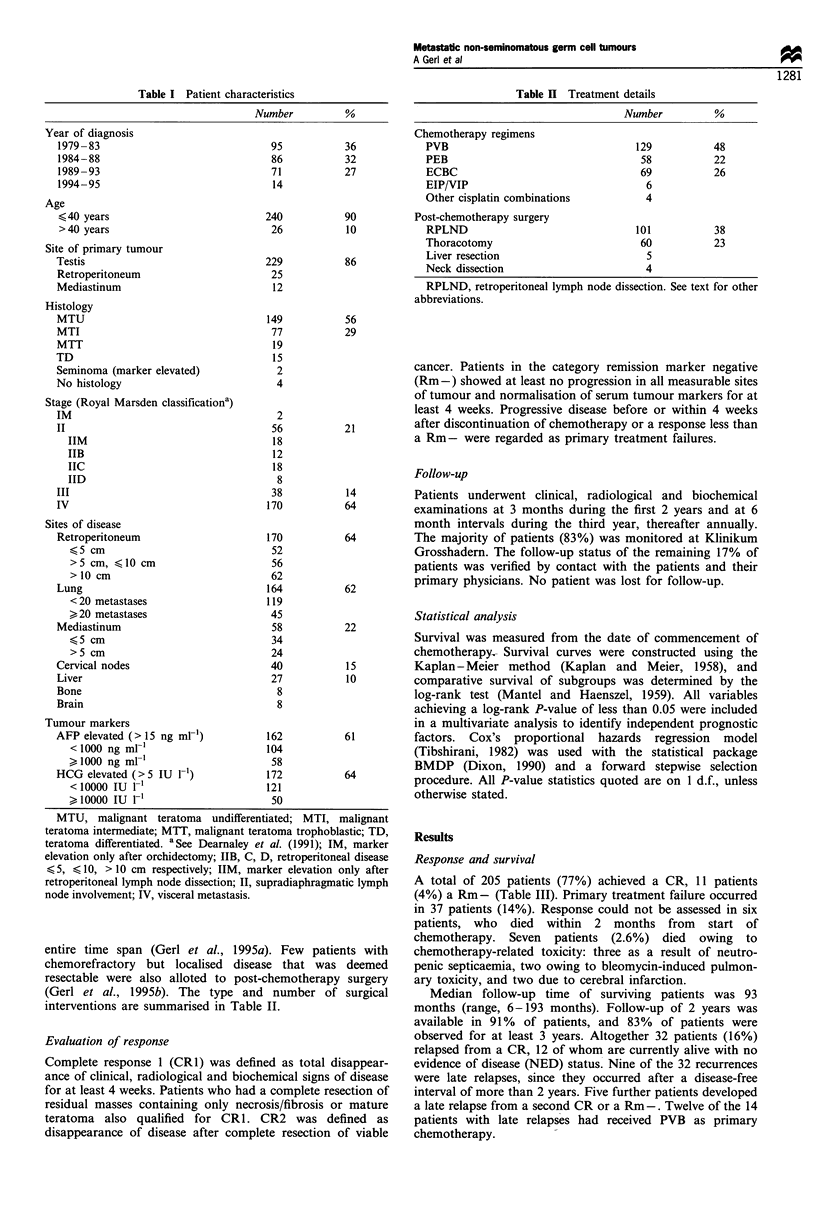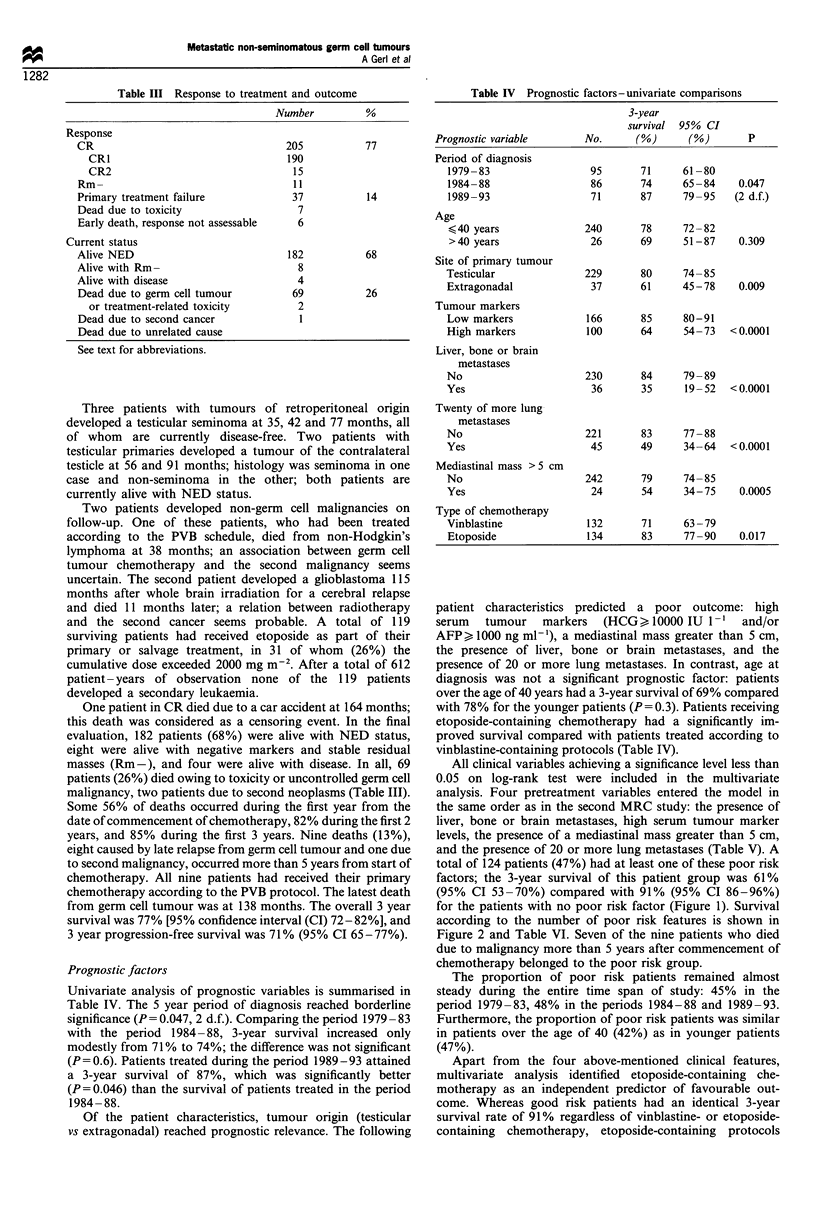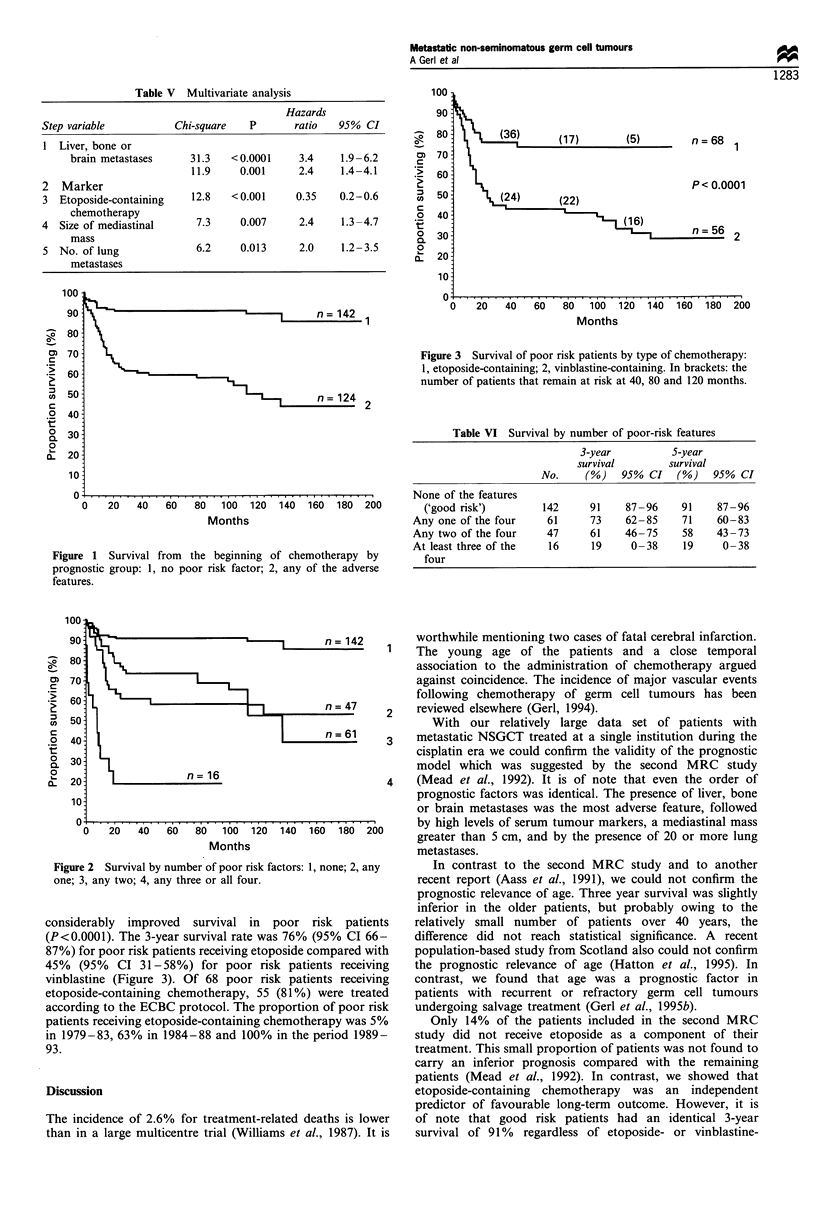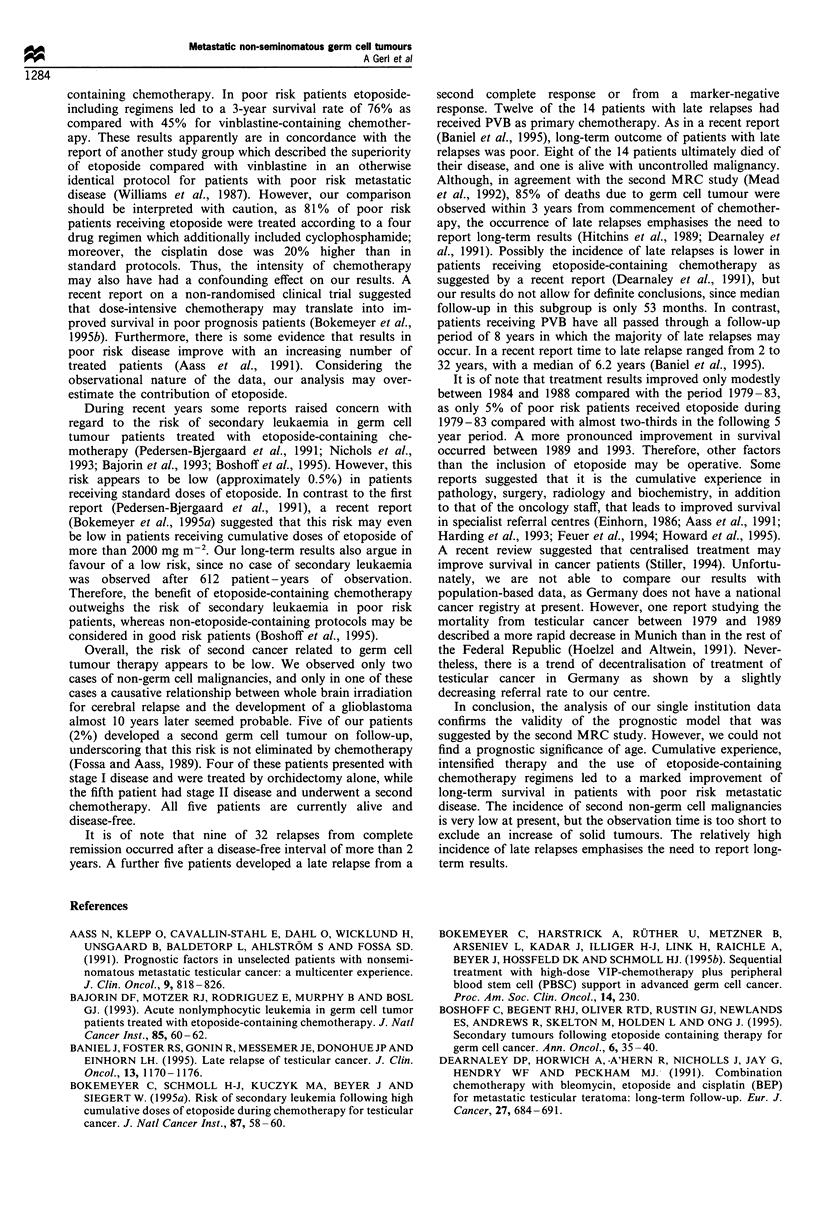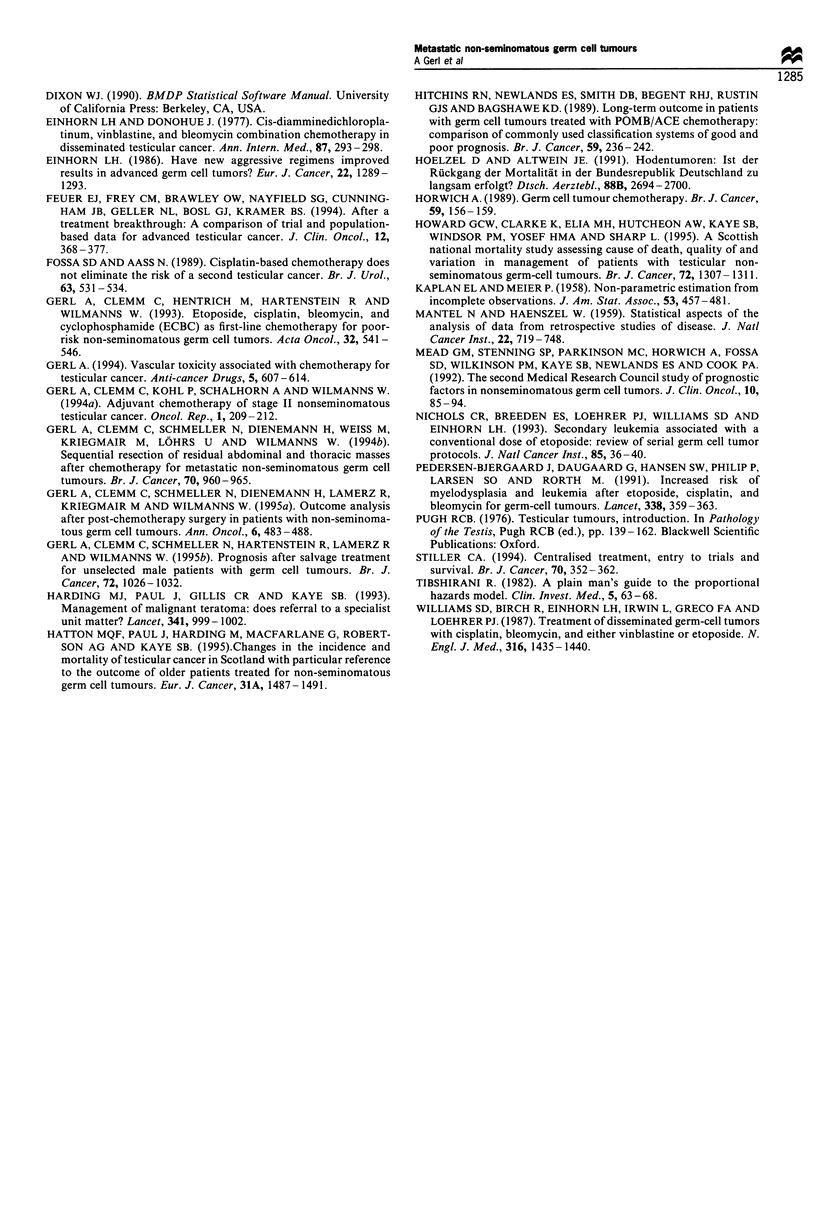# Advances in the management of metastatic non-seminomatous germ cell tumours during the cisplatin era: a single-institution experience.

**DOI:** 10.1038/bjc.1996.530

**Published:** 1996-10

**Authors:** A. Gerl, C. Clemm, N. Schmeller, R. Hartenstein, R. Lamerz, W. Wilmanns

**Affiliations:** Department of Internal Medicine III, Klinikum Grosshadern, University of Munich, Germany.

## Abstract

Long-term outcome was reviewed in 266 consecutive patients with metastatic non-seminomatous germ cell tumours treated at a single institution. The overall 3 year survival was 77%, and 3 year progression-free survival was 71%. Multivariate analysis identified the following clinical features as independent prognostic factors: the presence of liver, bone or brain metastasis, serum human chorionic gonadotropin > or = 10000 U l-1 and/or alpha-fetoprotein > or = 1000 ng ml-1, a mediastinal mass > 5 cm and the presence of 20 or more lung metastases. Age was not of prognostic significance. Patients without any of the above poor-risk factors had a 3 year survival of 91% regardless of etoposide- or vinblastine-containing chemotherapy compared with 61% for the remaining patients. However, etoposide-containing protocols led to significantly improved survival in patients with at least one poor risk factor. After 612 patient-years of observation no case of secondary leukaemia was observed among 119 surviving patients who had received etoposide as part of their treatment. With a median follow-up of 93 months, five patients developed a second germ cell tumour, two patients nongerm cell malignancies. Fourteen patients relapsed after a disease-free interval of more than 2 years, and nine patients died more than 5 years after commencement of treatment underscoring the need to report long-term results. There is some evidence that cumulative experience translates into improved survival and cure rates for patients with poor-risk metastatic disease.


British Journal of Cancer (1996) 74, 1280-1285
© 1996 Stockton Press All rights reserved 0007-0920/96 $12.00

### Advances in the management of metastatic non-seminomatous germ cell tumours during the cisplatin era: a single-institution experience

A Gerl[1], C Clemm[2], N Schmeller[3], R Hartenstein[4], R Lamerz[5] and W Wilmanns[1,6]

[1]*Department of Internal Medicine III, Klinikum Grosshadern of the University of Munich;* [3]*Department of Urology, Klinikum Grosshadern of the University of Munich;* [5]*Department of Internal Medicine II, Klinikum Grosshadern of the University of Munich;* [2]*Department of Internal Medicine, Clinic of Oncology, Bad Trissl;* [4]*Department of Internal Medicine IV, Munich Harlaching City Hospital;* [6]*GSF Forschungszentrum für Umwelt und Gesundheit, Munich, Germany.*

**Summary** Long-term outcome was reviewed in 266 consecutive patients with metastatic non-seminomatous germ cell tumours treated at a single institution. The overall 3 year survival was 77%, and 3 year progression-free survival was 71%. Multivariate analysis identified the following clinical features as independent prognostic factors: the presence of liver, bone or brain metastasis, serum human chorionic gonadotropin$\geqslant$10000 U $l^{-1}$ and/or alpha-fetoprotein$\geqslant$1000 ng $ml^{-1}$, a mediastinal mass>5 cm and the presence of 20 or more lung metastases. Age was not of prognostic significance. Patients without any of the above poor-risk factors had a 3-year survival of 91% regardless of etoposide- or vinblastine-containing chemotherapy compared with 61% for the remaining patients. However, etoposide-containing protocols led to significantly improved survival in patients with at least one poor risk factor. After 612 patient-years of observation no case of secondary leukaemia was observed among 119 surviving patients who had received etoposide as part of their treatment. With a median follow-up of 93 months, five patients developed a second germ cell tumour, two patients non-germ cell malignancies. Fourteen patients relapsed after a disease-free interval of more than 2 years, and nine patients died more than 5 years after commencement of treatment underscoring the need to report long-term results. There is some evidence that cumulative experience translates into improved survival and cure rates for patients with poor-risk metastatic disease.

**Keywords:** non-seminomatous germ cell tumour; chemotherapy; etoposide; prognosis; second neoplasm

Testicular cancer is the most common neoplasm in males aged under 40. Cisplatin-based combination chemotherapy has dramatically improved the clinical outcome of patients with metastatic non-seminomatous germ cell tumours (NSGCTs) (Einhorn and Donohue, 1977; Horwich, 1989). However, approximately 20% of patients with metastatic NSGCT still die of their disease. Recently, the second Medical Research Council (MRC) study including data from 795 patients with metastatic NSGCT from 13 centres defined a simple prognostic classification using four clinical features as prognostic variables. Whereas good-risk patients had a 3 year survival of 93%, patients with at least one of the adverse features had a 3 year survival rate of 67% (Mead *et al.*, 1992).

The present paper analyses our single institution experience in the management of metastatic NSGCT during the cisplatin era. The above-mentioned prognostic model is tested on our data set, and the prognostic relevance of age is assessed. Particular emphasis is put on whether the substitution of etoposide for vinblastine translates into an improved survival. Moreover, we describe the incidence of late relapses and second malignancies.

### Patients and methods

### *Patient characteristics*

A total of 266 consecutive patients with metastatic NSGCT underwent primary cisplatin-based chemotherapy at Klinikum Grosshadern between May 1979 and June 1995. Patients with stage IIA/B were predominantly treated surgically, the majority of whom received adjuvant chemotherapy. The latter patient group was not included in this study; results have been reported elsewhere (Gerl *et al.*, 1994*a*).

Correspondence: A Gerl, Medizinische Klinik III, Klinikum Grosshadern, Marchioninistrasse 15, 81377 München, Germany
Received 8 February 1996; revised 25 April 1996; accepted 7 May 1996

The median age at diagnosis was 27 years (range, 16-72 years). Histology was established according to the British Testicular Tumour Panel criteria (Pugh, 1976). No primary histology was available in four cases, but a considerable elevation of serum human chorionic gonadotropin (HCG) and/or alpha-fetoprotein (AFP) indicated the presence of NSGCT. Two patients had pure seminomas in their testicular primaries, but high levels of HCG (both cases) and AFP (one case) disclosed the presence of NSGCT. Prechemotherapy staging consisted of physical examination, laboratory testing including serum tumour marker determination, chest radiograph and abdominal and thoracic computerised tomography (CT) scans. Further examinations were performed as indicated by clinical symptoms. The characteristics of the 266 patients pertaining to the status immediately before initiation of chemotherapy are summarised in Table I.

### *Treatment*

Up to 1983 all patients received chemotherapy according to the PVB protocol consisting of cisplatin 20 mg $m^{-2}$ on days 1-5, vinblastine 0.15-0.20 mg $kg^{-1}$ on days 1 and 2, and bleomycin 30 mg on days 2, 9 and 16 (Einhorn and Donohue, 1977). Since the end of 1983 patients with a large tumour burden have been predominantly treated according to the ECBC regimen consisting of etoposide 120 mg $m^{-2}$ on days 1-4, cisplatin 30 mg $m^{-2}$ on days 1-4, bleomycin 15 mg on day 1 (bolus) and 12 mg $m^{-2}$ on days 1-4 (24 h infusion), and cyclophosphamide 300 mg $m^{-2}$ on days 1-4 (Gerl *et al.*, 1993). In 1987 we began to treat patients with low-volume metastatic disease according to the PEB protocol substituting etoposide 100 mg $m^{-2}$ on days 1-5 for vinblastine (Williams *et al.*, 1987). Few patients received cisplatin-ifosfamide-based chemotherapy with either vinblastine (VIP) or etoposide (EIP) or other cisplatin combinations (Table II). Patients who achieved normalisation of serum tumour markers but had radiographic abnormalities were alloted to adjunctive post-chemotherapy surgery. Some patients underwent multiple surgical interventions (Gerl *et al.*, 1994*b*). The resection rate remained steady during the

**Table I** Patient characteristics

| | *Number* | *%* |
|---|---|---|
| Year of diagnosis | | |
| 1979–83 | 95 | 36 |
| 1984–88 | 86 | 32 |
| 1989–93 | 71 | 27 |
| 1994–95 | 14 | |
| Age | | |
| ≤40 years | 240 | 90 |
| >40 years | 26 | 10 |
| Site of primary tumour | | |
| Testis | 229 | 86 |
| Retroperitoneum | 25 | |
| Mediastinum | 12 | |
| Histology | | |
| MTU | 149 | 56 |
| MTI | 77 | 29 |
| MTT | 19 | |
| TD | 15 | |
| Seminoma (marker elevated) | 2 | |
| No histology | 4 | |
| Stage (Royal Marsden classification[a]) | | |
| IM | 2 | |
| II | 56 | 21 |
| IIM | 18 | |
| IIB | 12 | |
| IIC | 18 | |
| IID | 8 | |
| III | 38 | 14 |
| IV | 170 | 64 |
| Sites of disease | | |
| Retroperitoneum | 170 | 64 |
| ≤5 cm | 52 | |
| >5 cm, ≤10 cm | 56 | |
| >10 cm | 62 | |
| Lung | 164 | 62 |
| <20 metastases | 119 | |
| ≥20 metastases | 45 | |
| Mediastinum | 58 | 22 |
| ≤5 cm | 34 | |
| >5 cm | 24 | |
| Cervical nodes | 40 | 15 |
| Liver | 27 | 10 |
| Bone | 8 | |
| Brain | 8 | |
| Tumour markers | | |
| AFP elevated (>15 ng $ml^{-1}$) | 162 | 61 |
| <1000 ng $ml^{-1}$ | 104 | |
| ≥1000 ng $ml^{-1}$ | 58 | |
| HCG elevated (>5 IU $l^{-1}$) | 172 | 64 |
| <10000 IU $l^{-1}$ | 121 | |
| ≥10000 IU $l^{-1}$ | 50 | |

MTU, malignant teratoma undifferentiated; MTI, malignant teratoma intermediate; MTT, malignant teratoma trophoblastic; TD, teratoma differentiated. [a]See Dearnaley *et al.* (1991); IM, marker elevation only after orchidectomy; IIB, C, D, retroperitoneal disease ≤5, ≤10, >10 cm respectively; IIM, marker elevation only after retroperitoneal lymph node dissection; II, supradiaphragmatic lymph node involvement; IV, visceral metastasis.

entire time span (Gerl *et al.*, 1995*a*). Few patients with chemorefractory but localised disease that was deemed resectable were also alloted to post-chemotherapy surgery (Gerl *et al.*, 1995*b*). The type and number of surgical interventions are summarised in Table II.

*Evaluation of response*

Complete response 1 (CR1) was defined as total disappearance of clinical, radiological and biochemical signs of disease for at least 4 weeks. Patients who had a complete resection of residual masses containing only necrosis/fibrosis or mature teratoma also qualified for CR1. CR2 was defined as disappearance of disease after complete resection of viable

**Table II** Treatment details

| | *Number* | *%* |
|---|---|---|
| Chemotherapy regimens | | |
| PVB | 129 | 48 |
| PEB | 58 | 22 |
| ECBC | 69 | 26 |
| EIP/VIP | 6 | |
| Other cisplatin combinations | 4 | |
| Post-chemotherapy surgery | | |
| RPLND | 101 | 38 |
| Thoracotomy | 60 | 23 |
| Liver resection | 5 | |
| Neck dissection | 4 | |

RPLND, retroperitoneal lymph node dissection. See text for other abbreviations.

cancer. Patients in the category remission marker negative (Rm–) showed at least no progression in all measurable sites of tumour and normalisation of serum tumour markers for at least 4 weeks. Progressive disease before or within 4 weeks after discontinuation of chemotherapy or a response less than a Rm– were regarded as primary treatment failures.

*Follow-up*

Patients underwent clinical, radiological and biochemical examinations at 3 months during the first 2 years and at 6 month intervals during the third year, thereafter annually. The majority of patients (83%) was monitored at Klinikum Grosshadern. The follow-up status of the remaining 17% of patients was verified by contact with the patients and their primary physicians. No patient was lost for follow-up.

*Statistical analysis*

Survival was measured from the date of commencement of chemotherapy. Survival curves were constructed using the Kaplan–Meier method (Kaplan and Meier, 1958), and comparative survival of subgroups was determined by the log-rank test (Mantel and Haenszel, 1959). All variables achieving a log-rank *P*-value of less than 0.05 were included in a multivariate analysis to identify independent prognostic factors. Cox's proportional hazards regression model (Tibshirani, 1982) was used with the statistical package BMDP (Dixon, 1990) and a forward stepwise selection procedure. All *P*-value statistics quoted are on 1 d.f., unless otherwise stated.

### Results

*Response and survival*

A total of 205 patients (77%) achieved a CR, 11 patients (4%) a Rm– (Table III). Primary treatment failure occurred in 37 patients (14%). Response could not be assessed in six patients, who died within 2 months from start of chemotherapy. Seven patients (2.6%) died owing to chemotherapy-related toxicity: three as a result of neutropenic septicaemia, two owing to bleomycin-induced pulmonary toxicity, and two due to cerebral infarction.

Median follow-up time of surviving patients was 93 months (range, 6–193 months). Follow-up of 2 years was available in 91% of patients, and 83% of patients were observed for at least 3 years. Altogether 32 patients (16%) relapsed from a CR, 12 of whom are currently alive with no evidence of disease (NED) status. Nine of the 32 recurrences were late relapses, since they occurred after a disease-free interval of more than 2 years. Five further patients developed a late relapse from a second CR or a Rm–. Twelve of the 14 patients with late relapses had received PVB as primary chemotherapy.

**Table III** Response to treatment and outcome

| | *Number* | *%* |
|---|---|---|
| Response | | |
| CR | 205 | 77 |
| CR1 | 190 | |
| CR2 | 15 | |
| Rm− | 11 | |
| Primary treatment failure | 37 | 14 |
| Dead due to toxicity | 7 | |
| Early death, response not assessable | 6 | |
| Current status | | |
| Alive NED | 182 | 68 |
| Alive with Rm− | 8 | |
| Alive with disease | 4 | |
| Dead due to germ cell tumour | 69 | 26 |
| or treatment-related toxicity | 2 | |
| Dead due to second cancer | 1 | |
| Dead due to unrelated cause | | |

See text for abbreviations.

**Table IV** Prognostic factors–univariate comparisons

| *Prognostic variable* | *No.* | *3-year survival (%)* | *95% CI (%)* | P |
|---|---|---|---|---|
| Period of diagnosis | | | | |
| 1979–83 | 95 | 71 | 61–80 | |
| 1984–88 | 86 | 74 | 65–84 | 0.047 |
| 1989–93 | 71 | 87 | 79–95 | (2 d.f.) |
| Age | | | | |
| ⩽40 years | 240 | 78 | 72–82 | |
| >40 years | 26 | 69 | 51–87 | 0.309 |
| Site of primary tumour | | | | |
| Testicular | 229 | 80 | 74–85 | |
| Extragonadal | 37 | 61 | 45–78 | 0.009 |
| Tumour markers | | | | |
| Low markers | 166 | 85 | 80–91 | |
| High markers | 100 | 64 | 54–73 | <0.0001 |
| Liver, bone or brain metastases | | | | |
| No | 230 | 84 | 79–89 | |
| Yes | 36 | 35 | 19–52 | <0.0001 |
| Twenty of more lung metastases | | | | |
| No | 221 | 83 | 77–88 | |
| Yes | 45 | 49 | 34–64 | <0.0001 |
| Mediastinal mass >5 cm | | | | |
| No | 242 | 79 | 74–85 | |
| Yes | 24 | 54 | 34–75 | 0.0005 |
| Type of chemotherapy | | | | |
| Vinblastine | 132 | 71 | 63–79 | |
| Etoposide | 134 | 83 | 77–90 | 0.017 |

Three patients with tumours of retroperitoneal origin developed a testicular seminoma at 35, 42 and 77 months, all of whom are currently disease-free. Two patients with testicular primaries developed a tumour of the contralateral testicle at 56 and 91 months; histology was seminoma in one case and non-seminoma in the other; both patients are currently alive with NED status.

Two patients developed non-germ cell malignancies on follow-up. One of these patients, who had been treated according to the PVB schedule, died from non-Hodgkin's lymphoma at 38 months; an association between germ cell tumour chemotherapy and the second malignancy seems uncertain. The second patient developed a glioblastoma 115 months after whole brain irradiation for a cerebral relapse and died 11 months later; a relation between radiotherapy and the second cancer seems probable. A total of 119 surviving patients had received etoposide as part of their primary or salvage treatment, in 31 of whom (26%) the cumulative dose exceeded 2000 mg $m^{-2}$. After a total of 612 patient–years of observation none of the 119 patients developed a secondary leukaemia.

One patient in CR died due to a car accident at 164 months; this death was considered as a censoring event. In the final evaluation, 182 patients (68%) were alive with NED status, eight were alive with negative markers and stable residual masses (Rm−), and four were alive with disease. In all, 69 patients (26%) died owing to toxicity or uncontrolled germ cell malignancy, two patients due to second neoplasms (Table III). Some 56% of deaths occurred during the first year from the date of commencement of chemotherapy, 82% during the first 2 years, and 85% during the first 3 years. Nine deaths (13%), eight caused by late relapse from germ cell tumour and one due to second malignancy, occurred more than 5 years from start of chemotherapy. All nine patients had received their primary chemotherapy according to the PVB protocol. The latest death from germ cell tumour was at 138 months. The overall 3 year survival was 77% [95% confidence interval (CI) 72–82%], and 3 year progression-free survival was 71% (95% CI 65–77%).

### *Prognostic factors*

Univariate analysis of prognostic variables is summarised in Table IV. The 5 year period of diagnosis reached borderline significance ($P=0.047$, 2 d.f.). Comparing the period 1979–83 with the period 1984–88, 3-year survival increased only modestly from 71% to 74%; the difference was not significant ($P=0.6$). Patients treated during the period 1989–93 attained a 3-year survival of 87%, which was significantly better ($P=0.046$) than the survival of patients treated in the period 1984–88.

Of the patient characteristics, tumour origin (testicular *vs* extragonadal) reached prognostic relevance. The following patient characteristics predicted a poor outcome: high serum tumour markers (HCG⩾10000 IU $l^{-1}$ and/or AFP⩾1000 ng $ml^{-1}$), a mediastinal mass greater than 5 cm, the presence of liver, bone or brain metastases, and the presence of 20 or more lung metastases. In contrast, age at diagnosis was not a significant prognostic factor: patients over the age of 40 years had a 3-year survival of 69% compared with 78% for the younger patients ($P=0.3$). Patients receiving etoposide-containing chemotherapy had a significantly improved survival compared with patients treated according to vinblastine-containing protocols (Table IV).

All clinical variables achieving a significance level less than 0.05 on log-rank test were included in the multivariate analysis. Four pretreatment variables entered the model in the same order as in the second MRC study: the presence of liver, bone or brain metastases, high serum tumour marker levels, the presence of a mediastinal mass greater than 5 cm, and the presence of 20 or more lung metastases (Table V). A total of 124 patients (47%) had at least one of these poor risk factors; the 3-year survival of this patient group was 61% (95% CI 53–70%) compared with 91% (95% CI 86–96%) for the patients with no poor risk factor (Figure 1). Survival according to the number of poor risk features is shown in Figure 2 and Table VI. Seven of the nine patients who died due to malignancy more than 5 years after commencement of chemotherapy belonged to the poor risk group.

The proportion of poor risk patients remained almost steady during the entire time span of study: 45% in the period 1979–83, 48% in the periods 1984–88 and 1989–93. Furthermore, the proportion of poor risk patients was similar in patients over the age of 40 (42%) as in younger patients (47%).

Apart from the four above-mentioned clinical features, multivariate analysis identified etoposide-containing chemotherapy as an independent predictor of favourable outcome. Whereas good risk patients had an identical 3-year survival rate of 91% regardless of vinblastine- or etoposide-containing chemotherapy, etoposide-containing protocols

**Table V** Multivariate analysis

| *Step variable* | *Chi-square* | P | *Hazards ratio* | *95% CI* |
|---|---|---|---|---|
| 1 Liver, bone or brain metastases | 31.3 | <0.0001 | 3.4 | 1.9–6.2 |
| | 11.9 | 0.001 | 2.4 | 1.4–4.1 |
| 2 Marker | | | | |
| 3 Etoposide-containing chemotherapy | 12.8 | <0.001 | 0.35 | 0.2–0.6 |
| 4 Size of mediastinal mass | 7.3 | 0.007 | 2.4 | 1.3–4.7 |
| 5 No. of lung metastases | 6.2 | 0.013 | 2.0 | 1.2–3.5 |

**Figure 1** Survival from the beginning of chemotherapy by prognostic group: 1, no poor risk factor; 2, any of the adverse features.

**Figure 2** Survival by number of poor risk factors: 1, none; 2, any one; 3, any two; 4, any three or all four.

considerably improved survival in poor risk patients ($P<0.0001$). The 3-year survival rate was 76% (95% CI 66–87%) for poor risk patients receiving etoposide compared with 45% (95% CI 31–58%) for poor risk patients receiving vinblastine (Figure 3). Of 68 poor risk patients receiving etoposide-containing chemotherapy, 55 (81%) were treated according to the ECBC protocol. The proportion of poor risk patients receiving etoposide-containing chemotherapy was 5% in 1979–83, 63% in 1984–88 and 100% in the period 1989–93.

### Discussion

The incidence of 2.6% for treatment-related deaths is lower than in a large multicentre trial (Williams *et al.*, 1987). It is

**Figure 3** Survival of poor risk patients by type of chemotherapy: 1, etoposide-containing; 2, vinblastine-containing. In brackets: the number of patients that remain at risk at 40, 80 and 120 months.

**Table VI** Survival by number of poor-risk features

| | *No.* | *3-year survival (%)* | *95% CI* | *5-year survival (%)* | *95% CI* |
|---|---|---|---|---|---|
| None of the features ('good risk') | 142 | 91 | 87–96 | 91 | 87–96 |
| Any one of the four | 61 | 73 | 62–85 | 71 | 60–83 |
| Any two of the four | 47 | 61 | 46–75 | 58 | 43–73 |
| At least three of the four | 16 | 19 | 0–38 | 19 | 0–38 |

worthwhile mentioning two cases of fatal cerebral infarction. The young age of the patients and a close temporal association to the administration of chemotherapy argued against coincidence. The incidence of major vascular events following chemotherapy of germ cell tumours has been reviewed elsewhere (Gerl, 1994).

With our relatively large data set of patients with metastatic NSGCT treated at a single institution during the cisplatin era we could confirm the validity of the prognostic model which was suggested by the second MRC study (Mead *et al.*, 1992). It is of note that even the order of prognostic factors was identical. The presence of liver, bone or brain metastases was the most adverse feature, followed by high levels of serum tumour markers, a mediastinal mass greater than 5 cm, and by the presence of 20 or more lung metastases.

In contrast to the second MRC study and to another recent report (Aass *et al.*, 1991), we could not confirm the prognostic relevance of age. Three year survival was slightly inferior in the older patients, but probably owing to the relatively small number of patients over 40 years, the difference did not reach statistical significance. A recent population-based study from Scotland also could not confirm the prognostic relevance of age (Hatton *et al.*, 1995). In contrast, we found that age was a prognostic factor in patients with recurrent or refractory germ cell tumours undergoing salvage treatment (Gerl *et al.*, 1995*b*).

Only 14% of the patients included in the second MRC study did not receive etoposide as a component of their treatment. This small proportion of patients was not found to carry an inferior prognosis compared with the remaining patients (Mead *et al.*, 1992). In contrast, we showed that etoposide-containing chemotherapy was an independent predictor of favourable long-term outcome. However, it is of note that good risk patients had an identical 3-year survival of 91% regardless of etoposide- or vinblastinecontaining chemotherapy. In poor risk patients etoposide-including regimens led to a 3-year survival rate of 76% as compared with 45% for vinblastine-containing chemotherapy. These results apparently are in concordance with the report of another study group which described the superiority of etoposide compared with vinblastine in an otherwise identical protocol for patients with poor risk metastatic disease (Williams *et al*., 1987). However, our comparison should be interpreted with caution, as 81% of poor risk patients receiving etoposide were treated according to a four drug regimen which additionally included cyclophosphamide; moreover, the cisplatin dose was 20% higher than in standard protocols. Thus, the intensity of chemotherapy may also have had a confounding effect on our results. A recent report on a non-randomised clinical trial suggested that dose-intensive chemotherapy may translate into improved survival in poor prognosis patients (Bokemeyer *et al*., 1995*b*). Furthermore, there is some evidence that results in poor risk disease improve with an increasing number of treated patients (Aass *et al*., 1991). Considering the observational nature of the data, our analysis may overestimate the contribution of etoposide.

During recent years some reports raised concern with regard to the risk of secondary leukaemia in germ cell tumour patients treated with etoposide-containing chemotherapy (Pedersen-Bjergaard *et al*., 1991; Nichols *et al*., 1993; Bajorin *et al*., 1993; Boshoff *et al*., 1995). However, this risk appears to be low (approximately 0.5%) in patients receiving standard doses of etoposide. In contrast to the first report (Pedersen-Bjergaard *et al*., 1991), a recent report (Bokemeyer *et al*., 1995*a*) suggested that this risk may even be low in patients receiving cumulative doses of etoposide of more than 2000 mg $m^{-2}$. Our long-term results also argue in favour of a low risk, since no case of secondary leukaemia was observed after 612 patient–years of observation. Therefore, the benefit of etoposide-containing chemotherapy outweighs the risk of secondary leukaemia in poor risk patients, whereas non-etoposide-containing protocols may be considered in good risk patients (Boshoff *et al*., 1995).

Overall, the risk of second cancer related to germ cell tumour therapy appears to be low. We observed only two cases of non-germ cell malignancies, and only in one of these cases a causative relationship between whole brain irradiation for cerebral relapse and the development of a glioblastoma almost 10 years later seemed probable. Five of our patients (2%) developed a second germ cell tumour on follow-up, underscoring that this risk is not eliminated by chemotherapy (Fossa and Aass, 1989). Four of these patients presented with stage I disease and were treated by orchidectomy alone, while the fifth patient had stage II disease and underwent a second chemotherapy. All five patients are currently alive and disease-free.

It is of note that nine of 32 relapses from complete remission occurred after a disease-free interval of more than 2 years. A further five patients developed a late relapse from a second complete response or from a marker-negative response. Twelve of the 14 patients with late relapses had received PVB as primary chemotherapy. As in a recent report (Baniel *et al*., 1995), long-term outcome of patients with late relapses was poor. Eight of the 14 patients ultimately died of their disease, and one is alive with uncontrolled malignancy. Although, in agreement with the second MRC study (Mead *et al*., 1992), 85% of deaths due to germ cell tumour were observed within 3 years from commencement of chemotherapy, the occurrence of late relapses emphasises the need to report long-term results (Hitchins *et al*., 1989; Dearnaley *et al*., 1991). Possibly the incidence of late relapses is lower in patients receiving etoposide-containing chemotherapy as suggested by a recent report (Dearnaley *et al*., 1991), but our results do not allow for definite conclusions, since median follow-up in this subgroup is only 53 months. In contrast, patients receiving PVB have all passed through a follow-up period of 8 years in which the majority of late relapses may occur. In a recent report time to late relapse ranged from 2 to 32 years, with a median of 6.2 years (Baniel *et al*., 1995).

It is of note that treatment results improved only modestly between 1984 and 1988 compared with the period 1979–83, as only 5% of poor risk patients received etoposide during 1979–83 compared with almost two-thirds in the following 5 year period. A more pronounced improvement in survival occurred between 1989 and 1993. Therefore, other factors than the inclusion of etoposide may be operative. Some reports suggested that it is the cumulative experience in pathology, surgery, radiology and biochemistry, in addition to that of the oncology staff, that leads to improved survival in specialist referral centres (Einhorn, 1986; Aass *et al*., 1991; Harding *et al*., 1993; Feuer *et al*., 1994; Howard *et al*., 1995). A recent review suggested that centralised treatment may improve survival in cancer patients (Stiller, 1994). Unfortunately, we are not able to compare our results with population-based data, as Germany does not have a national cancer registry at present. However, one report studying the mortality from testicular cancer between 1979 and 1989 described a more rapid decrease in Munich than in the rest of the Federal Republic (Hoelzel and Altwein, 1991). Nevertheless, there is a trend of decentralisation of treatment of testicular cancer in Germany as shown by a slightly decreasing referral rate to our centre.

In conclusion, the analysis of our single institution data confirms the validity of the prognostic model that was suggested by the second MRC study. However, we could not find a prognostic significance of age. Cumulative experience, intensified therapy and the use of etoposide-containing chemotherapy regimens led to a marked improvement of long-term survival in patients with poor risk metastatic disease. The incidence of second non-germ cell malignancies is very low at present, but the observation time is too short to exclude an increase of solid tumours. The relatively high incidence of late relapses emphasises the need to report long-term results.

### References

AASS N, KLEPP O, CAVALLIN-STAHL E, DAHL O, WICKLUND H, UNSGAARD B, BALDETORP L, AHLSTRÖM S AND FOSSA SD. (1991). Prognostic factors in unselected patients with nonseminomatous metastatic testicular cancer: a multicenter experience. *J. Clin. Oncol*., **9**, 818–826.

BAJORIN DF, MOTZER RJ, RODRIGUEZ E, MURPHY B AND BOSL GJ. (1993). Acute nonlymphocytic leukemia in germ cell tumor patients treated with etoposide-containing chemotherapy. *J. Natl Cancer Inst*., **85**, 60–62.

BANIEL J, FOSTER RS, GONIN R, MESSEMER JE, DONOHUE JP AND EINHORN LH. (1995). Late relapse of testicular cancer. *J. Clin. Oncol*., **13**, 1170–1176.

BOKEMEYER C, SCHMOLL H-J, KUCZYK MA, BEYER J AND SIEGERT W. (1995*a*). Risk of secondary leukemia following high cumulative doses of etoposide during chemotherapy for testicular cancer. *J. Natl Cancer Inst*., **87**, 58–60.

BOKEMEYER C, HARSTRICK A, RÜTHER U, METZNER B, ARSENIEV L, KADAR J, ILLIGER H-J, LINK H, RAICHLE A, BEYER J, HOSSFELD DK AND SCHMOLL HJ. (1995*b*). Sequential treatment with high-dose VIP-chemotherapy plus peripheral blood stem cell (PBSC) support in advanced germ cell cancer. *Proc. Am. Soc. Clin. Oncol*., **14**, 230.

BOSHOFF C, BEGENT RHJ, OLIVER RTD, RUSTIN GJ, NEWLANDS ES, ANDREWS R, SKELTON M, HOLDEN L AND ONG J. (1995). Secondary tumours following etoposide containing therapy for germ cell cancer. *Ann. Oncol*., **6**, 35–40.

DEARNALEY DP, HORWICH A, A'HERN R, NICHOLLS J, JAY G, HENDRY WF AND PECKHAM MJ. (1991). Combination chemotherapy with bleomycin, etoposide and cisplatin (BEP) for metastatic testicular teratoma: long-term follow-up. *Eur. J. Cancer*, **27**, 684–691.

DIXON WJ. (1990). *BMDP Statistical Software Manual.* University of California Press: Berkeley, CA, USA.

EINHORN LH AND DONOHUE J. (1977). Cis-diamminedichloroplatinum, vinblastine, and bleomycin combination chemotherapy in disseminated testicular cancer. *Ann. Intern. Med.*, **87**, 293–298.

EINHORN LH. (1986). Have new aggressive regimens improved results in advanced germ cell tumors? *Eur. J. Cancer*, **22**, 1289–1293.

FEUER EJ, FREY CM, BRAWLEY OW, NAYFIELD SG, CUNNINGHAM JB, GELLER NL, BOSL GJ, KRAMER BS. (1994). After a treatment breakthrough: A comparison of trial and population-based data for advanced testicular cancer. *J. Clin. Oncol.*, **12**, 368–377.

FOSSA SD AND AASS N. (1989). Cisplatin-based chemotherapy does not eliminate the risk of a second testicular cancer. *Br. J. Urol.*, **63**, 531–534.

GERL A, CLEMM C, HENTRICH M, HARTENSTEIN R AND WILMANNS W. (1993). Etoposide, cisplatin, bleomycin, and cyclophosphamide (ECBC) as first-line chemotherapy for poor-risk non-seminomatous germ cell tumors. *Acta Oncol.*, **32**, 541–546.

GERL A. (1994). Vascular toxicity associated with chemotherapy for testicular cancer. *Anti-cancer Drugs*, **5**, 607–614.

GERL A, CLEMM C, KOHL P, SCHALHORN A AND WILMANNS W. (1994*a*). Adjuvant chemotherapy of stage II nonseminomatous testicular cancer. *Oncol. Rep.*, **1**, 209–212.

GERL A, CLEMM C, SCHMELLER N, DIENEMANN H, WEISS M, KRIEGMAIR M, LÖHRS U AND WILMANNS W. (1994*b*). Sequential resection of residual abdominal and thoracic masses after chemotherapy for metastatic non-seminomatous germ cell tumours. *Br. J. Cancer*, **70**, 960–965.

GERL A, CLEMM C, SCHMELLER N, DIENEMANN H, LAMERZ R, KRIEGMAIR M AND WILMANNS W. (1995*a*). Outcome analysis after post-chemotherapy surgery in patients with non-seminomatous germ cell tumours. *Ann. Oncol.*, **6**, 483–488.

GERL A, CLEMM C, SCHMELLER N, HARTENSTEIN R, LAMERZ R AND WILMANNS W. (1995*b*). Prognosis after salvage treatment for unselected male patients with germ cell tumours. *Br. J. Cancer*, **72**, 1026–1032.

HARDING MJ, PAUL J, GILLIS CR AND KAYE SB. (1993). Management of malignant teratoma: does referral to a specialist unit matter? *Lancet*, **341**, 999–1002.

HATTON MQF, PAUL J, HARDING M, MACFARLANE G, ROBERTSON AG AND KAYE SB. (1995).Changes in the incidence and mortality of testicular cancer in Scotland with particular reference to the outcome of older patients treated for non-seminomatous germ cell tumours. *Eur. J. Cancer*, **31A**, 1487–1491.

HITCHINS RN, NEWLANDS ES, SMITH DB, BEGENT RHJ, RUSTIN GJS AND BAGSHAWE KD. (1989). Long-term outcome in patients with germ cell tumours treated with POMB/ACE chemotherapy: comparison of commonly used classification systems of good and poor prognosis. *Br. J. Cancer*, **59**, 236–242.

HOELZEL D AND ALTWEIN JE. (1991). Hodentumoren: Ist der Rückgang der Mortalität in der Bundesrepublik Deutschland zu langsam erfolgt? *Dtsch. Aerztebl.*, **88B**, 2694–2700.

HORWICH A. (1989). Germ cell tumour chemotherapy. *Br. J. Cancer*, **59**, 156–159.

HOWARD GCW, CLARKE K, ELIA MH, HUTCHEON AW, KAYE SB, WINDSOR PM, YOSEF HMA AND SHARP L. (1995). A Scottish national mortality study assessing cause of death, quality of and variation in management of patients with testicular non-seminomatous germ-cell tumours. *Br. J. Cancer*, **72**, 1307–1311.

KAPLAN EL AND MEIER P. (1958). Non-parametric estimation from incomplete observations. *J. Am. Stat. Assoc.*, **53**, 457–481.

MANTEL N AND HAENSZEL W. (1959). Statistical aspects of the analysis of data from retrospective studies of disease. *J. Natl Cancer Inst.*, **22**, 719–748.

MEAD GM, STENNING SP, PARKINSON MC, HORWICH A, FOSSA SD, WILKINSON PM, KAYE SB, NEWLANDS ES AND COOK PA. (1992). The second Medical Research Council study of prognostic factors in nonseminomatous germ cell tumors. *J. Clin. Oncol.*, **10**, 85–94.

NICHOLS CR, BREEDEN ES, LOEHRER PJ, WILLIAMS SD AND EINHORN LH. (1993). Secondary leukemia associated with a conventional dose of etoposide: review of serial germ cell tumor protocols. *J. Natl Cancer Inst.*, **85**, 36–40.

PEDERSEN-BJERGAARD J, DAUGAARD G, HANSEN SW, PHILIP P, LARSEN SO AND RORTH M. (1991). Increased risk of myelodysplasia and leukemia after etoposide, cisplatin, and bleomycin for germ-cell tumours. *Lancet*, **338**, 359–363.

PUGH RCB. (1976). Testicular tumours, introduction. In *Pathology of the Testis*, Pugh RCB (ed.), pp. 139–162. Blackwell Scientific Publications: Oxford.

STILLER CA. (1994). Centralised treatment, entry to trials and survival. *Br. J. Cancer*, **70**, 352–362.

TIBSHIRANI R. (1982). A plain man's guide to the proportional hazards model. *Clin. Invest. Med.*, **5**, 63–68.

WILLIAMS SD, BIRCH R, EINHORN LH, IRWIN L, GRECO FA AND LOEHRER PJ. (1987). Treatment of disseminated germ-cell tumors with cisplatin, bleomycin, and either vinblastine or etoposide. *N. Engl. J. Med.*, **316**, 1435–1440.